# ﻿Different observers introduce not negligible biases in comparative karyomorphological studies

**DOI:** 10.3897/compcytogen.18.135172

**Published:** 2024-09-24

**Authors:** Lorenzo Peruzzi, Jacopo Franzoni, Manuel Tiburtini, Emanuela Abidi, Emiliano Alù, Giulio Barone, Elisabetta Bianchi, Chiara Cataudella, Emanuela Di Iorio, Maria Guerrina, Fabio Mondello, Luca Paino, Mario Pentassuglia, Manuela Porrovecchio, Giovanni Rivieccio, Eugenia Siccardi, Adriano Stinca, Alessio Tei, Virginia Volanti, Antonio Giacò

**Affiliations:** 1 PLANTSEED Lab, Department of Biology, University of Pisa, Pisa, Italy; 2 Department of Agricultural, Forest and Food Sciences, University of Palermo, Palermo, Italy; 3 Department of Biology, University of Firenze, Firenze, Italy; 4 Department of Biology, University of Napoli “Federico II”, Botanical Garden, Napoli, Italy; 5 DISTAV, University of Genova, Genova, Italy; 6 Department of Chemical, Biological, Pharmaceutical, and Environmental Sciences, University of Messina, Messina, Italy; 7 Department of Biological, Geological and Environmental Sciences, University of Catania, Catania, Italy; 8 Department of Chemical, Physical, Mathematical and Natural Sciences, University of Sassari, Sassari, Italy; 9 Department of Environmental, Biological and Pharmaceutical Sciences and Technologies, University of Campania Luigi Vanvitelli, Caserta, Italy; 10 Department of Agriculture, Food and Environment, University of Pisa, Pisa, Italy

**Keywords:** Cytogenetics, cytosystematics, cytotaxonomy, karyotype asymmetry, karyotype structure

## Abstract

Within a practical course of cytotaxonomy organized in Pisa (Italy) on February 2024 by the Group for Floristics, Systematics and Evolution of the Italian Botanical Society, we tested whether using image analysis softwares possible biases are still introduced by different observers. We conclude that observer bias selectively applies in possibly overestimating the length of short arms in a karyotype. As a consequence, the parameters most sensitive to these possible errors are CV_CI_ and CV_CL_, and to a less degree M_CA_ and THL. To achieve more stable results among observers, a still lacking standardized measurement protocol could be helpful.

## ﻿Introduction

Karyomorphology is an easy, cheap and powerful approach to obtain useful basic comparative information in systematic studies (Astuti et al. 2017). This usually implies the measurement of chromosomes (i.e. length of long arm [L], short arm [S], and other derived information) in spread metaphase plates, to describe the phenotypic aspect of the chromosome complement (Levin 2002; Guerra 2012). The most commonly used traits to characterize a karyotype structure and asymmetry are: the chromosome number (2*n*), the basic chromosome number (*x*), the total haploid (monoploid) chromosome length (THL), the mean centromeric asymmetry (M_CA_), the coefficient of variation of chromosome length (CV_CL_), and the coefficient of variation of centromeric index (CV_CI_) (Peruzzi and Altınordu 2014).

However, while obtaining the chromosome number and basic chromosome number (see also Peruzzi 2013) should be a relatively easy task, it is well known that the reliability of karyomorphological measurements can be influenced by two main causes (Sybenga 1959; Bentzer et al. 1971): a) variation in actual chromosome length, b) variation caused by inaccuracy of the measurement. The first cause is biological and linked to several phenomena, which may alter the degree of chromosome condensation (e.g., Bentzer et al. 1971; Mártonfiová 2013; Mehravi et al. 2022; Franzoni et al. 2024). The second cause of variation is “artificial” and pertains to variation in methods and observer (Sybenga 1959; Essad et al. 1966; Bentzer et al. 1971). In particular, Bentzer et al. (1971) also addressed the question whether the same measurements made by different people produce consistent data, and showed that this is not the case, especially using camera lucida drawings of metaphase plates. Starting from the early 2000s, a new era of chromosome measurement through image analysis softwares started (e.g., Rasband 1997 onwards, Mirzaghaderi and Marzangi 2015; Altınordu et al. 2016; Kirov et al. 2017; Liu et al. 2023; Stossi and Singh 2023), certainly making the measurements more accurate than in the twentieth century, when scholars were forcedly based on camera lucida drawings or printed microphotographs. However, no information is available whether using image analysis softwares possible biases are still introduced by different observers in measuring the very same microphotographs.

We addressed this problem within a practical course organized in Pisa (Italy) between 6 and 9 February 2024 by the Group for Floristics, Systematics and Evolution of the Italian Botanical Society.

## ﻿Material and methods

**Figure 1.**
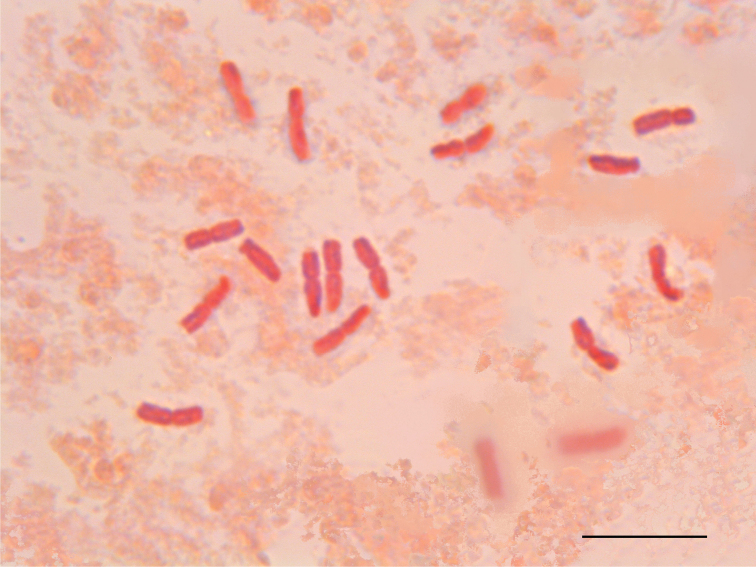
The metaphase plate of Santolinadecumbenssubsp.diversifolia (from Giacò et al. 2022) distributed to the participants for independent measurements. The image was built by pasting several images at different focus, in order to be able to see all the 2*n* = 18 chromosomes in the same picture. Scale bar: 10 µm.

A metaphase plate of the diploid (2*n* = 18) angiosperm Santolinadecumbens Miller, 1768 subsp. diversifolia (Jordan et Fourreau,1869) Giacò et Peruzzi, 2022 (Asteraceae; Giacò et al. 2023) obtained from plants collected in Sisteron, Provence-Alpes-Côte d’Azur, France (Fig. 1) was taken from those used in the work by Giacò et al. (2022). This metaphase plate was given to all the participants to the course, who independently measured it, by using the software MATO (Liu et al. 2023).

We focused on the following quantitative traits (Peruzzi and Altınordu 2014; Astuti et al. 2017):

- THL (total haploid [monoploid] length of chromosome complement). It is a gross proxy of genome size (Carta and Peruzzi 2016; Franzoni et al. 2024), and is obtained by the sum of the length of all the chromosomes in a metaphase plate, divided by the ploidy level.
- M _CA_ (mean centromeric asymmetry). It expresses the intrachromosomal karyotype asymmetry (Peruzzi and Eroğlu 2013), and is calculated as the mean value of the difference between the two (complementary) proportions L/(L+S) and S/(L+S), multiplied by 100.
- CV _CL_ (coefficient of variation of chromosome length). It expresses the interchromosomal karyotype asymmetry (Paszko 2006), and is calculated as the standard deviation of chromosome lengths (L+S) in a complement, divided by the mean chromosome length and multiplied by 100.
- CV _CI_ (coefficient of variation of centromeric index). It expresses the degree of heterogeneity in the position of centromere in a karyotype (Zuo and Yuan 2011), and is calculated as the standard deviation of centromeric index S/(L+S) in a complement, divided by the mean centromeric index and multiplied by 100.

The variation of each karyomorphological trait cited above was illustrated by means of boxplots. Then, to test which karyomorphological traits are more prone to biases introduced by different observers, for each trait a CV was calculated. Finally, correlations between parameters were tested by Pearson’s correlation coefficient. All the analyses have been carried out in PAST 4.17 (Hammer et al. 2001; Hammer 2024).

## ﻿Results

**Figure 2.**
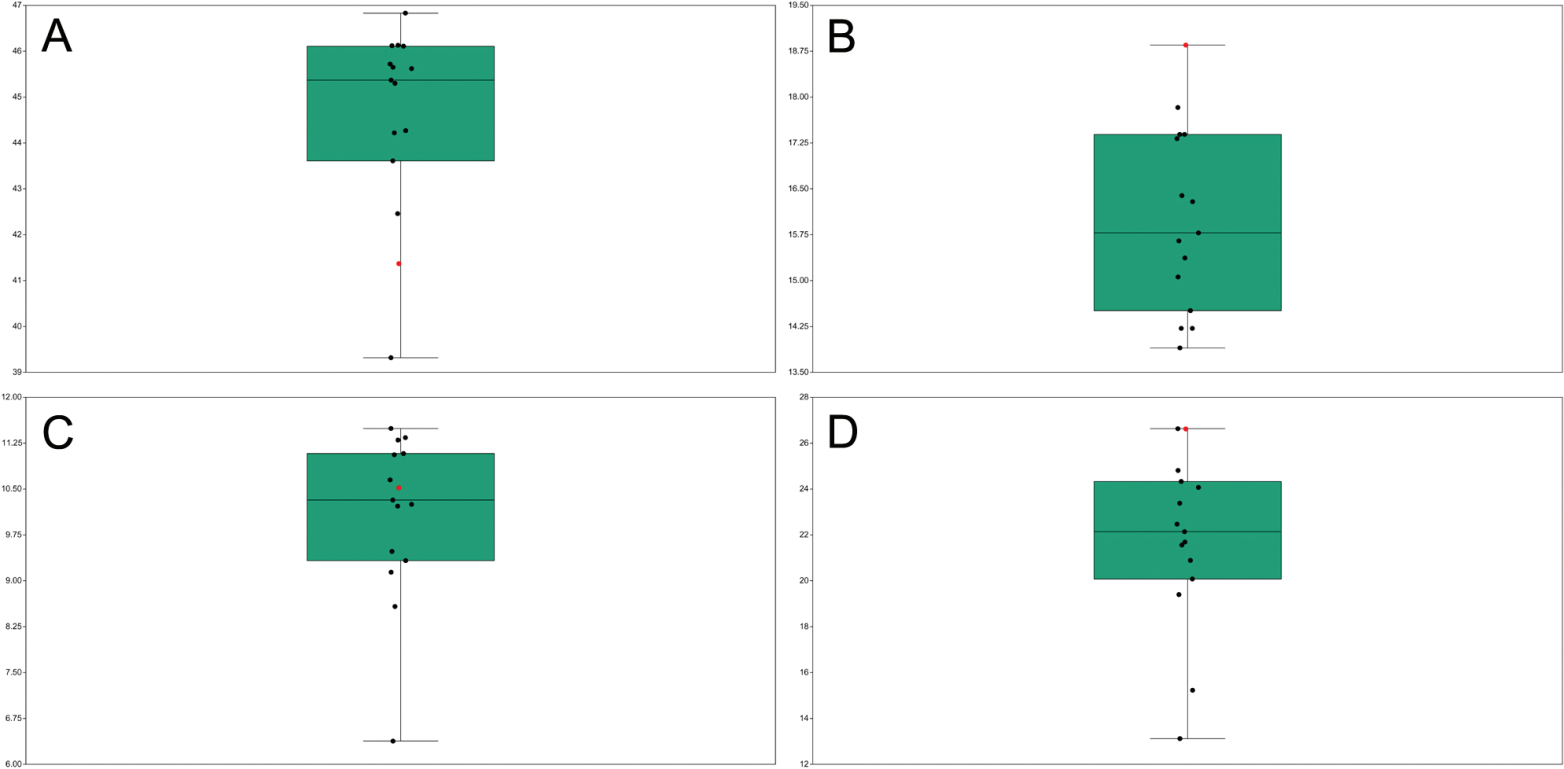
Boxplots with jitters illustrating the variability in the karyomorphological traits THL (A), M_CA_ (B), CV_CL_ (C), and CV_CI_ (D) independently calculated by the participants based on the same metaphase plate of Santolinadecumbenssubsp.diversifolia in Fig. 1. The red dot is the measurement n. 15 (see Suppl. material 1: table S1), used to build the karyotype of this population by Giacò et al. (2022).

The variation of each karyomorphological trait is illustrated in Fig. 2, which is based on the data reported in Suppl. material 1: table S1. The coefficients of variation show the highest value concerning CV_CI_ (17.3%) and CV_CL_ (13.4%), and the lowest for M_CA_ (4.7%) and THL (9.4%).

**Figure 3.**
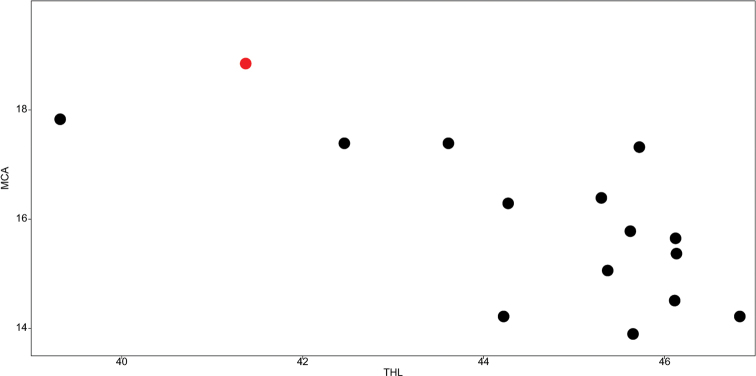
Scatter plot THL (x axis) vs. M_CA_ (y axis), highlighting the significant negative correlation among these two karyomorphological traits. The red dot is the measurement n. 15 (see Suppl. material 1: table S1), used to build the karyotype of this population by Giacò et al. (2022).

According to Table 1, the only highly statistically significant (p < 0.01) and negative correlation is between THL and M_CA_ (Fig. 3). A positive correlation between CV_CL_ and CV_CI_ is only marginally significant (p < 0.05), while all other correlations are not significant (p > 0.05).

**Table 1.** Pearson’s correlation coefficients and p values among the considered quantitative karyomorphological traits based on 15 measurements independently made by different evaluators on the same metaphase plate of Santolinadecumbenssubsp.diversifolia. In bold are highlighted the significant correlations.

	THL	M_CA_	CV_CL_	CV_CI_
THL		**p = 0.0022365**	p = 0.40465	p = 0.055789
M_CA_	-**0.7248**		p = 0.38316	p = 0.056293
CV_CL_	-0.23236	+0.38316		**p = 0.010211**
CV_CI_	-0.50333	+0.63978	+**0.50225**	

## ﻿Discussion

The significant negative correlation between THL and M_CA_ points towards selective observer bias that tends to overestimate the length of the short arm. Indeed, such an overestimation could at the same time cause an increment of THL and a decrease in MCA. Indeed, already Sybenga (1959) and Bentzer et al. (1971) evidenced how possible measurement errors can become of increasing importance in case of small chromosomes / small chromosome arms. Possibly, the same correlation is not found in CV_CI_ because this parameter is based on centromeric index [S/(L+S)], so that an overestimation of short arm would have consequences both at the numerator and at the denominator of the centromeric index. On one side, this causes the lack of correlation between CV_CI_ and THL, while on the other side it causes a lot of further variation in this parameter, which is the most subjected to observer bias (up to 17.3% in our experiment). These errors may be due to the different decisions made when selecting the centromere, as no standardized protocol has ever been proposed.

We can conclude that, in karyomorphology, observer bias selectively applies in possibly overestimating the length of short arms in a karyotype. As a consequence, the parameters most sensitive to these possible errors are CV_CI_ and CV_CL_, and to a less degree M_CA_ and THL.

Accordingly, we recommend special attention in recognizing and measuring correctly the short arms of chromosomes, which are the main source of observer bias in cytogenetics. To achieve this, a homogeneous approach among observers could be helpful. Moreover, the motto already claimed by Bentzer et al. (1971) “*in the course of an investigation all the measurements should be made by the same person*” also fully applies to the era of image analysis.

## ﻿Author contributions

Lorenzo Peruzzi – methodology, Lorenzo Peruzzi – validation, Antonio Giacò, Emanuela Abidi, Emiliano Alù, Giulio Barone, Elisabetta Bianchi, Chiara Cataudella, Emanuela Di Iorio, Maria Guerrina, Fabio Mondello, Luca Paino, Mario Pentassuglia, Manuela Porrovecchio, Giovanni Rivieccio, Eugenia Siccardi, Adriano Stinca, Alessio Tei, Virginia Volanti – investigation, Lorenzo Peruzzi – writing and original draft preparation, Jacopo Franzoni, Antonio Giacò, Manuel Tiburtini – writing – review and editing, Lorenzo Peruzzi – funding acquisition. All authors have read and agreed to the published version of the manuscript.

## ﻿ORCID

**Lorenzo Peruzzi** https://orcid.org/0000-0001-9008-273X

**Jacopo Franzoni** https://orcid.org/0000-0002-0888-8396

**Manuel Tiburtini** https://orcid.org/0000-0003-4985-3058

**Giulio Barone** https://orcid.org/0000-0002-6345-3117

**Elisabetta Bianchi** https://orcid.org/0000-0003-1197-4081

**Fabio Mondello** https://orcid.org/0000-0002-9522-6277

**Manuela Porrovecchio** https://orcid.org/0009-0009-2349-4975

**Giovanni Rivieccio** https://orcid.org/0000-0003-0840-0212

**Eugenia Siccardi** https://orcid.org/0009-0008-4738-0633

**Adriano Stinca** https://orcid.org/0000-0002-8275-0184

**Alessio Tei** https://orcid.org/0009-0001-1326-609X

## Supplementary materials

Supplementary material 1 Karyomorphological traits Data type docx Explanation note **table S1.** Karyomorphological traits independently calculated by the participants based on the same metaphase plate of Santolinadecumbenssubsp.diversifolia in Fig. 1. https://binary.pensoft.net/file/1140697This dataset is made available under the Open Database License (http://opendatacommons.org/licenses/odbl/1.0/). The Open Database License (ODbL) is a license agreement intended to allow users to freely share, modify, and use this Dataset while maintaining this same freedom for others, provided that the original source and author(s) are credited.Lorenzo Peruzzi, Jacopo Franzoni, Manuel Tiburtini, Emanuela Abidi, Emiliano Alù, Giulio Barone, Elisabetta Bianchi, Chiara Cataudella, Emanuela Di Iorio, Maria Guerrina, Fabio Mondello, Luca Paino, Mario Pentassuglia, Manuela Porrovecchio, Giovanni Rivieccio, Eugenia Siccardi, Adriano Stinca, Alessio Tei, Virginia Volanti, Antonio Giacò
