# Supplementary material for: ﻿Different observers introduce not negligible biases in comparative karyomorphological studies
Source: Comp Cytogenet. 2024 Sep 24;18:175–82. doi: 10.3897/compcytogen.18.135172 (PMC11445605; doi:10.3897/compcytogen.18.135172)
Supplement: Supplementary material 1 — Karyomorphological traits [file comparative_cytogenetics-18-175_article-135172__-s001.docx]

**Table S1**. Karyomorphological traits independently calculated by the participants based on the same metaphase plate of *Santolina decumbens* subsp. *diversifolia* in Fig. 1.

| Observer | 2*n* | *x* | THL (µm) | M_CA_ | CV_CL_ | CV_CI_ |
| --- | --- | --- | --- | --- | --- | --- |
| 1 | 18 | 9 | 43.61 | 17.39 | 10.32 | 23.38 |
| 2 | 18 | 9 | 45.62 | 15.78 | 10.25 | 24.07 |
| 3 | 18 | 9 | 45.72 | 17.32 | 10.65 | 22.47 |
| 4 | 18 | 9 | 45.3 | 16.39 | 8.58 | 21.56 |
| 5 | 18 | 9 | 45.65 | 13.9 | 6.38 | 13.12 |
| 6 | 18 | 9 | 46.13 | 15.37 | 11.3 | 21.69 |
| 7 | 18 | 9 | 46.11 | 14.51 | 11.08 | 20.89 |
| 8 | 18 | 9 | 44.22 | 14.22 | 11.06 | 24.33 |
| 9 | 18 | 9 | 46.83 | 14.22 | 11.34 | 20.08 |
| 10 | 18 | 9 | 45.37 | 15.06 | 9.14 | 24.81 |
| 11 | 18 | 9 | 42.46 | 17.39 | 10.22 | 22.14 |
| 12 | 18 | 9 | 39.32 | 17.83 | 11.49 | 26.63 |
| 13 | 18 | 9 | 46.12 | 15.65 | 9.48 | 19.4 |
| 14 | 18 | 9 | 44.27 | 16.29 | 9.33 | 15.23 |
| 15* | 18 | 9 | 41.37 | 18.85 | 10.52 | 26.62 |

* measurements of the plate from Giacò et al. (2022).
